# Targeting the heparin-binding domain of fibroblast growth factor receptor 1 as a potential cancer therapy

**DOI:** 10.1186/s12943-015-0391-4

**Published:** 2015-07-23

**Authors:** Ling Ling, Si Kee Tan, Ting Hwee Goh, Edwin Cheung, Victor Nurcombe, Andre J. van Wijnen, Simon M. Cool

**Affiliations:** Institute of Medical Biology, Agency for Science, Technology and Research (A*STAR), 8A Biomedical Grove, #06-06 Immunos, Singapore, 138648 Singapore; Genome Institute of Singapore, Agency for Science, Technology and Research (A*STAR), 60 Biopolis Street, #02-01 Genome, Singapore, 138672 Singapore; Faculty of Health Sciences, University of Macau, E12 Avenida da Universidade, Taipa, Macau, China; Department of Orthopaedic Surgery, Yong Loo Lin School of Medicine, National University of Singapore, Singapore, 119074 Singapore; Department of Orthopedic Surgery & Biochemistry and Molecular Biology, Mayo Clinic, 200 First Street SW, MedSci 3-69, Rochester, MN 55905 USA

**Keywords:** Targeted therapy, Tumor, Cell death, Cell growth, Neutralizing antibody, Heparan sulfate

## Abstract

**Background:**

Aberrant activation of fibroblast growth factor receptors (FGFRs) deregulates cell proliferation and promotes cell survival, and may predispose to tumorigenesis. Therefore, selective inactivation of FGFRs is an important strategy for cancer therapy. Here as a proof-of-concept study, we developed a FGFR1 neutralizing antisera, IMB-R1, employing a novel strategy aimed at preventing the access of essential heparan sulfate (HS) co-receptors to the heparin-binding domain on FGFR1.

**Methods:**

The mRNA and protein expression level of FGFR1 and other FGFRs were examined in several lines of breast cancer and osteosarcoma cells and corresponding normal cells using Taqman real-time quantitative PCR and Western blot analysis. The specificity of IMB-R1 against FGFR1 was assessed with various ELISA-based approaches and Receptor Tyrosine Kinase array. Proliferation assay and apoptosis analysis were performed to assess the effect of IMB-R1 on cancer cell growth and apoptosis, respectively, in comparison with known FGFR1 inhibitors. The IMB-R1 induced alteration of intracellular signaling and gene expression were analysed using Western blot and microarray approaches. Immunohistochemical staining of FGFR1 using IMB-R1 were carried out in different cancer tissues from clinical patients. Throughout the study, statistical differences were determined by Student’s *t* test where appropriate and reported when a *p* value was less than 0.05.

**Results:**

We demonstrate that IMB-R1 is minimally cross-reactive for other FGFRs, and that it potently and specifically inhibits binding of heparin to FGFR1. Furthermore, IMB-R1 blocks the interaction of FGF2 with FGFR1, the kinase activity of FGFR1 and activation of intracellular FGFR signaling. Cancer cells treated with IMB-R1 displayed impaired FGF2 signaling, were unable to grow and instead underwent apoptosis. IMB-R1-induced cell death correlated with a disruption of antioxidative defense networks and increased expression of several tumor suppressors and apoptotic proteins, including p53. Immunostaining with IMB-R1 was stronger in human cancer tissues in which the FGFR1 gene is amplified.

**Conclusion:**

Our study suggests that blocking HS interaction with the heparin-binding domains of FGFR1 inhibited cancer cell growth, which can be an attractive strategy to inactivate cancer-related heparin-binding proteins.

**Electronic supplementary material:**

The online version of this article (doi:10.1186/s12943-015-0391-4) contains supplementary material, which is available to authorized users.

## Background

Cancer is the leading cause of death worldwide, accounting for around 13 % of all fatalities, with mortality numbers continuing to rise. Cancer therapy is one of the fastest growing segments in the pharmaceutical industry, which itself is undergoing a major shift from conventional chemotherapy and radiotherapy to specific agents aimed at targeting abnormalities that are specific to cancer cells. Amongst these are dysregulations of crucial growth factor cascades.

Fibroblast growth factors (FGFs) are pivotal regulators to key cellular processes, and their deregulation can lead to excessive proliferation, evasion of apoptosis, invasive behavior as well as aberrant angiogenic responses, all hallmarks of cancer. FGFs and their receptors (FGFRs) have been shown to be oncogenes in mouse tumor models [1] and their over-expression or over-activation have been observed in many cancers [2–4]. Of the over 500 pathways that have been implicated in oncogenic cascades, the kinases in FGF signaling are the most commonly mutated [5]. FGFs and FGFRs have thus become important targets for cancer intervention.

Research laboratories and pharmaceutical companies have been developing FGFR tyrosine kinase inhibitors (TKIs), antibodies against FGFRs and inactive receptor decoys as targeted cancer therapeutics. Several of the TKIs are undergoing clinical trials. However, most of these compounds are nucleotide analogs that have multiple targets due to similarity in structure of the intracellular kinase domains of FGFRs with vascular endothelial growth factor receptors (VEGFRs) and platelet-derived growth factor receptors (PDGFRs), as well as off-target inhibition of other proteins. Thus, recent efforts have focused on developing more specific FGFR TKIs, as well as neutralizing antibodies that are more specific than chemical inhibitors, particularly antibodies that target individual FGFR isoforms, so avoiding pan-FGFR inhibition [6]. Antibodies against FGFRs have been reported to inhibit tumor growth in xenograft models [7–9].

The family of FGFRs, consisting of four members (FGFRs1-4), are highly conserved, sharing 55–72 % amino acid sequence with each other [10]. FGFRs1 - 3 are widely expressed in adult human tissues, with FGFR4 exhibiting more limited tissue distribution [11]. The extracellular region of most FGFRs contains three immunoglobulin-like motifs (Ig domains). Alternative splicing of FGFRs1-3 mRNA transcripts within the IgIII domain results in two isoforms, IIIb and IIIc, which have altered affinity for FGFs and exhibit tissue-specific expression. The IIIb isoform is expressed mainly in epithelium and the IIIc in mesenchyme [12]. A heparin-binding domain, essential for interactions with heparin/heparan sulfate (HS), is located between IgII and IgIII [13]. FGFs are known to bind to the IgII and IgIII domains on FGFRs [14], triggering dimerization that subsequently activates intracellular tyrosine kinase activity and downstream signaling.

Ubiquitously distributed on cell surfaces and within the pericellular and extracellular matrix (ECM), HS is a highly complex molecule consisting of varying lengths of sulfated polysaccharides, some of which regulate growth factor signaling in a relatively specific manner through association with heparin-binding domains located on the ligand and/or receptor [15, 16]. HS is required to stabilize the FGF-FGFR interaction [17, 18] through the formation of an FGF:HS:FGFR ternary complex [14, 19].

Active mutations of FGFR2 or FGFR3 are common in various cancers [20], whereas the oncogenic impetus of FGFR1 results mainly from either gene amplification or overexpression of wild-type receptor [21–23]. FGFR1 overactivation occurs frequently in breast [24] and prostate cancer [25], with changes also reported in oral squamous carcinoma, ovarian cancer and bladder cancer [15, 26, 27]. In this work we hypothesized that FGFR1 signaling might be most effectively inhibited by blocking its interaction with HS. An antibody (IMB-R1) was thus raised to mask the heparin-binding domain on FGFR1. It proved to be particularly effective in suppressing FGF signaling, inducing strong apoptosis in cancer cells with tissue-specific potency. Our study supports the idea that FGFs/FGFRs are crucial for the survival of many cancer cells, and that targeting FGFR1 is a rational and effective strategy for therapy. More importantly, a novel strategy has been opened up for the targeted therapy of many other growth factor/receptors, that of preventing heparan sulfate/protein interactions.

## Results

### Increases in FGFR1 mRNA and protein expression in cancer cells

**Fig. 1:**
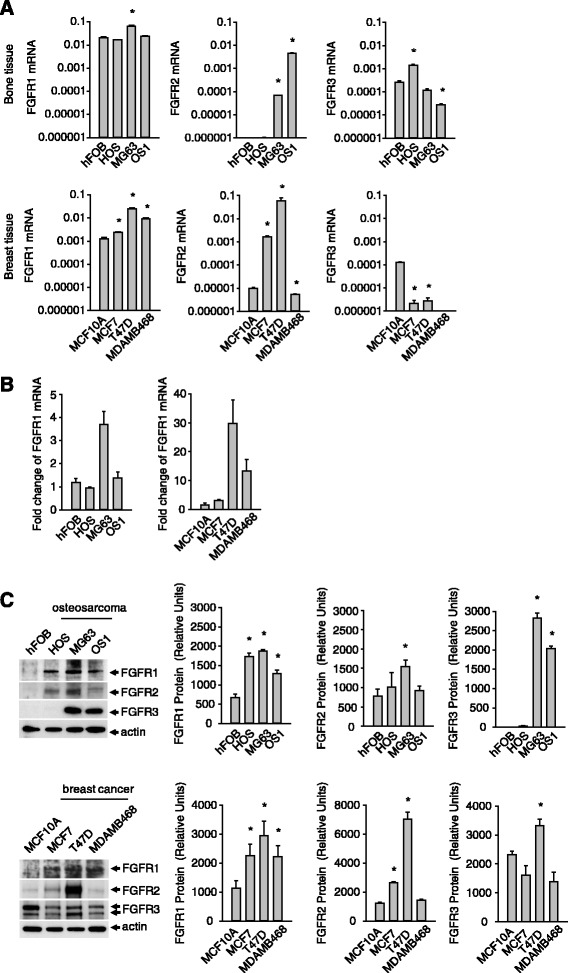
Expression of FGFR1 in cells from breast and bone tissue. **a**, FGFR mRNA transcript levels in normal and cancer cells. **b**, Fold change in FGFR mRNA based on expression in cancers cells relative to normal cells. **c**, FGFR protein levels in normal versus cancer cells. Results are from triplicate experiments and the Western blot is representative of the triplicates. (*p < 0.05)

**Fig. 2:**
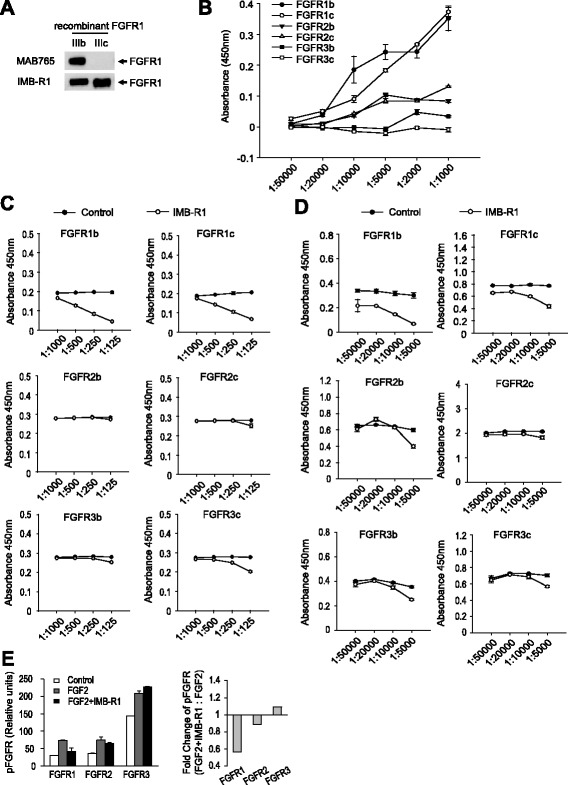
Specificity of IMB-R1 for FGFR1. **a**, Binding of IMB-R1 and MAB765 to FGFR1 isoforms (representative blot from triplicate experiments). **b**, Affinity of IMB-R1 for FGFR isoforms (results are from triplicate experiments). **c**, Binding of FGFR to heparin in the presence or absence of IMB-R1 (results are from triplicate experiments). **d**, Binding of FGF2 to FGFR in the presence or absence of IMB-R1 (results are from triplicate experiments). **e**, FGFR phosphorylation stimulated by FGF2 in the presence or absence of IMB-R1 including the fold change of FGFR phosphorylation as determined by a comparison of means

**Fig. 3:**
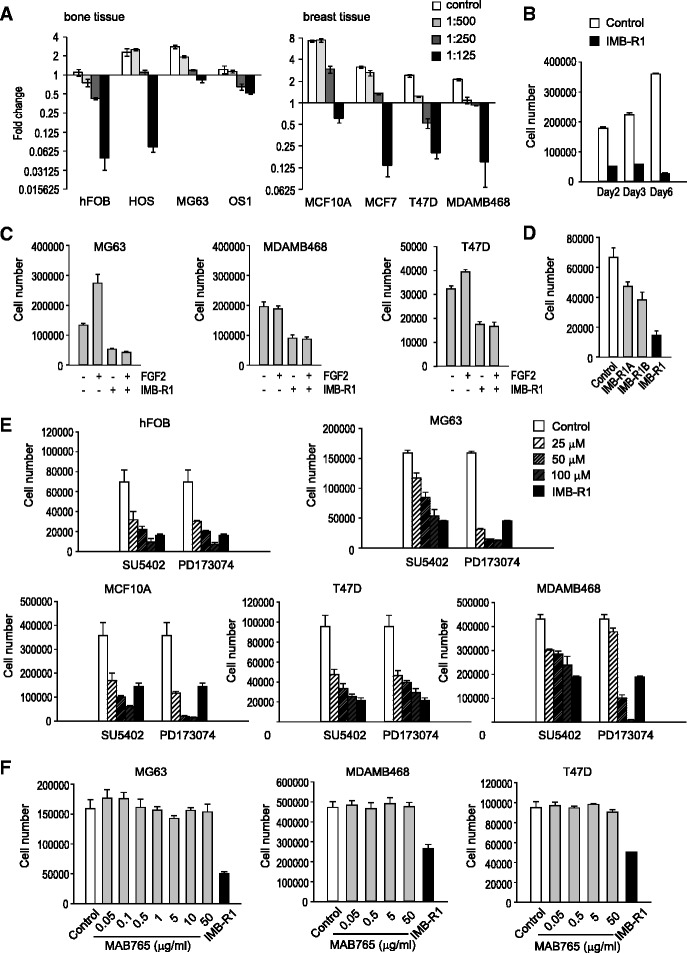
Effects of FGFR1 inhibitors on cell growth. **a**, Fold change in cell number 48 h after treatment with IMB-R1. **b**, Temporal change in MG63 cell number following treatment with IMB-R1 (1:250). **c**, Cell number following FGF2 treatment (20 ng/ml for MG63, 5 ng/ml for MDAMB468 and T47D) for 48 h. Cells were pre-treatment with IMB-R1 for 1 h before FGF2 treatment. **d**, Cell number (MG63) following 48 h treatment with IMB-R1 or antigen-purified IMB-R1. **e**, Cell numbers following 48 h treatment with varying doses of FGFR inhibitors. **f**, Cell numbers following 48 h treatment with the FGFR1 antibody (MAB765) at varying doses. Unless otherwise stated, IMB-R1 was applied at a dilution of 1:250. All experiments were performed in triplicate

**Fig. 4:**
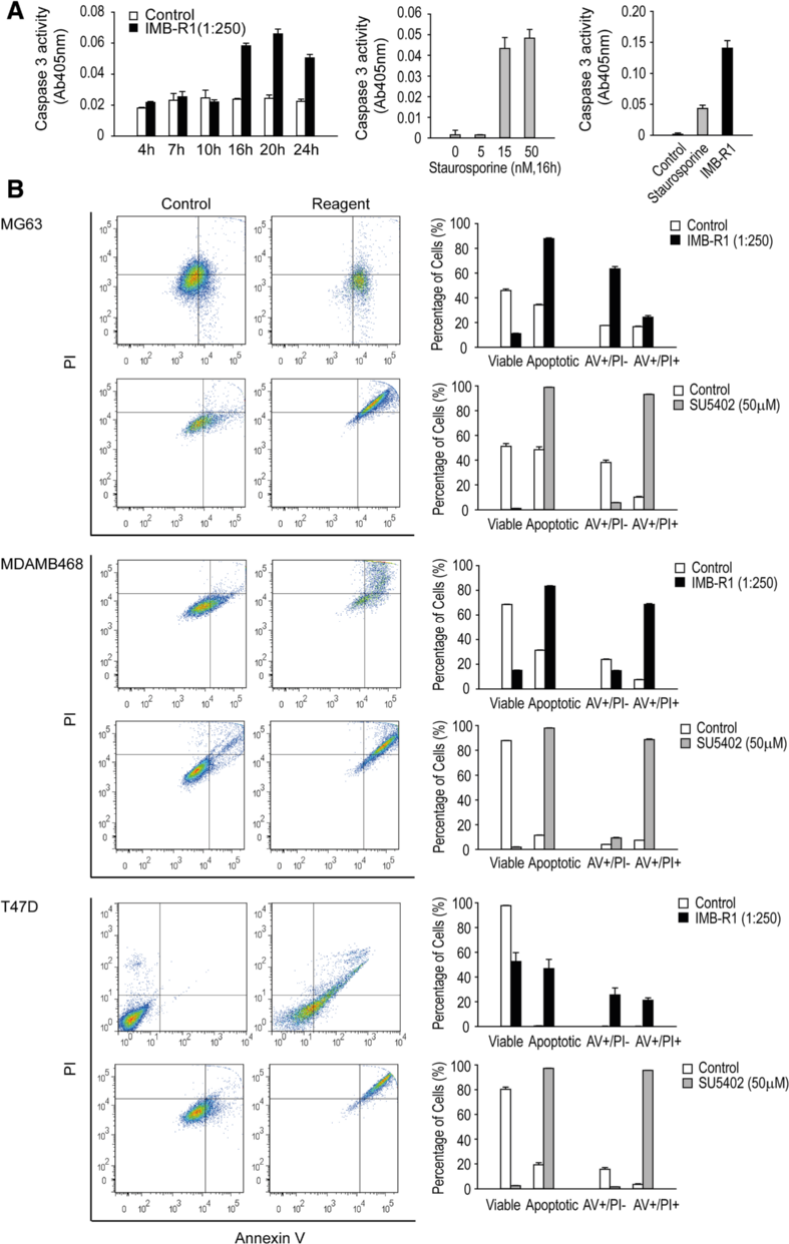
IMB-R1 induced cell apoptosis with varying potency. **a**, Caspase 3 activity was used as an assay for apoptosis in MG63 cells and the apoptotic activity of IMB-R1 (1:250) compared with Staurosporine (15 nM). **b**, Cells were treated with either IMB-R1 or SU5402 for 24 h and stained with a combination of Annexin V-FITC and PI to detect early apoptosis (positive for Annexin V-FITC, AV+/PI-) and late apoptosis (positive for both Annexin V-FITC and PI, AV+/PI+). Total apoptotic level represents AV+/PI- plus AV+/PI+. FACS plots are representative of triplicate experiments, and bar graphs show percentage cells in each population from triplicate experiments

Previous studies have indicated that FGFRs are over-expressed in cancer cells [28]. To check for expression in cancers of both epithelial and mesenchymal origin, we screened the expression of FGFR1-3 in human breast cancer and osteosarcoma cell lines as well as their normal counterparts—human mammary epithelial cells (MCF10A) and human fetal osteoblasts (hFOBs). FGFR1 mRNA transcripts were highly abundant in all cells tested, and significantly more (~10-fold) than other FGFRs except in T47D cells, where FGFR2 mRNA was detectable in slightly greater amounts (Fig. 1a). Comparing these FGFR1 levels with those in normal cells, we observed a significant increase in MG63 (~3-fold) and up-regulation in all 3 breast cancer lines (Fig. 1a and 1b). MG63 expressed the greatest levels of FGFR1 transcripts of all the cells tested (Fig. 1a), and was thus selected as the representative cancer cell model in subsequent assays (Fig. 2e, 3b, 3d and 4a). Importantly, FGFR1 protein was relatively overexpressed in all cancer cells tested as compared with normal cells (Fig. 1c). FGFR2 and FGFR3 were also up-regulated in some of the cancer cells, but not as markedly as FGFR1 (Fig. 1c).

### IMB-R1 specially bound and neutralized the activity of FGFR1

The confirmation that FGFR1 is over-expressed in cancer cells prompted us to develop a neutralizing antibody for FGFR1 intervention. Many studies have indicated that the heparin-binding domains (HBD) within FGFRs are important for FGF-FGFR interaction and subsequent cell signaling [17, 18]. Thus we aimed to design an antibody, designated here as IMB-R1, to mask the HBD of FGFR1 in order to compromise the ligand/receptor interaction (Additional file 1: Figure S1A). The 15 amino acid sequence immediately upstream of the HBD was selected as it both bears minimal homology to other FGFRs, and is highly hydrophilic, and thus exposed when the protein is in its native conformation [29]. FGFRs have multiple splice variants, with the most common isoforms IIIb (b) and IIIc (c) [30]. IMB-R1 recognized both isoforms, unlike the commercially available neutralizing antibody (MAB765), which only recognizes the b isoform (Fig. 2a). IMB-R1 exhibited specificity for FGFR1 in an ELISA assay employing recombinant human FGFR proteins. The affinity of IMB-R1 for FGFR1 dose-dependently increased (irrespective of isoform), in contrast to its relatively low affinity for FGFR2 and R3 (Fig. 2b). At the maximal dilution (1:1000), the affinity of IMB-R1 to FGFR1 was ~ 3-fold higher than FGFR2 and ~29-fold higher than FGFR3. The affinity of IMB-R1 for the FGFRs is at the ratio of: FGFR1:FGFR2:FGFR3 = 29:9:1.

To verify that IMB-R1 prevented HS from docking, its influence on heparin’s ability to bind directly to FGFRs was assessed in a protein-GAG binding assay. IMB-R1 potently prevented the heparin-FGFR1 interaction, but only slightly inhibited heparin binding to the other FGFRs (Fig. 2c). At the highest dose (1:125), 67–78 % of the heparin-FGFR1 interaction was blocked but less than 10 % of heparin-FGFR2 interaction and 27 % of the heparin-FGFR3c interaction (Additional file 1: Figure S1B).

We next examined whether IMB-R1 could neutralize FGFR1 activity. The effect of IMB-R1 on the FGF2-FGFR1 interaction was assessed by ELISA; IMB-R1 inhibited FGF2 binding by up to 77 % to the FGFR1b isoform and 43 % to the FGFR1c isoform (Fig. 2d, Additional file 1: Figure S1C). Notably, the effect of IMB-R1 on FGFR1 was more pronounced than on other FGFRs. FGFR phosphorylation was further examined in MG63 cells. IMB-R1 again reduced FGF2-stimulated phosphorylation of FGFR1 by 42 %, but only slightly (≈10 %) affected that of FGFR2 or FGFR3 (Fig. 2e). The enhanced phosphorylation of FGFR3 in the presence of IMB-R1 was presumably due to a compensation for the depressed FGFR1 signaling.

### IMB-R1 inhibited basal and FGF2 stimulated cell growth

Inhibition of FGFR1 should block FGF-induced mitogenic activity [31]; here IMB-R1 significantly inhibited cell growth in a dose-dependent manner. At the median dosage (1:250), IMB-R1 blocked growth in all the tested cells, and wherever the fold change was less than 1, cell numbers were less than those initially plated, suggesting cell death (Fig. 3a). IMB-R1 was more detrimental to the growth of normal osteoblasts (≥1:500 dilution) than osteosarcoma cells (≥1:250 dilution). In contrast, it took higher doses of IMB-R1 to kill normal breast cells (≥1:125 dilution) than breast cancer cells (≥1:250 dilution). Thus, normal mammary gland cells can tolerate greater amounts of IMB-R1 than cancer cells, with the opposite being true for bone tissue. In all cells tested, the IC_50_ was at least 1:250 dilution, and this dosage was used for subsequent studies. To eliminate a whole population requires a regimen of more than 6 days (Fig. 3b).

**Fig. 5:**
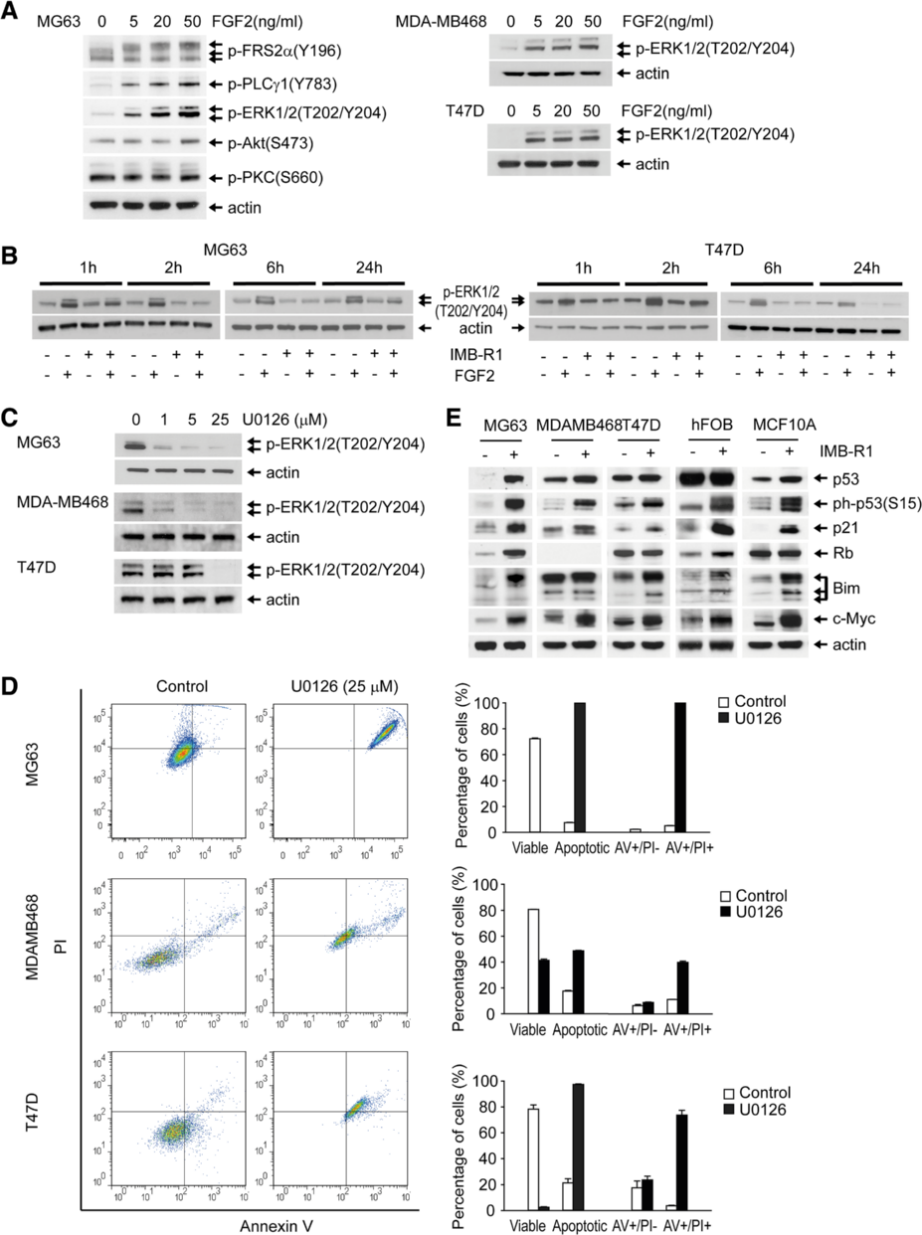
Downstream signaling targets of IMB-R1. Western blot analysis of signaling targets in serum-starved (SS) cells (48 h) released into FGF2 in the absence (**a**) or presence (**b**) of IMB-R1 for the time points indicated. **a**, Following SS, cells were stimulated with FGF2 for 10 min and protein expression determined. **b**, Following SS, cells were pre-treated with IMB-R1 (1:250 dilution) for 1, 2, 6 or 24 h then stimulated with 20 ng/ml (MG63) or 5 ng/ml (T47D) of FGF2 for 10 min and protein expression determined. **c**, Cells were dosed with U0126 for 1 h and phosphorylated-ERKs detected by immunoblotting. **d**, Cells were treated with U0126 for 24 h and stained with a combination of Annexin V-FITC and PI and presented as per Figure 4 **b**. **e**, Cells were treated with IMB-R1 (1:250 dilution) for 48 h and the protein expression determined by immunoblotting

Further assessment demonstrated that IMB-R1 blocked FGF2-stimulated cell proliferation. FGF2 dosage was pre-determined as per Figure 5a. FGF2 increased cell proliferation rates in MG63 and T47D, and IMB-R1 completely abrogated them (Fig. 3c). IMB-R1 inhibited the basal proliferation of MDAMB468 cells; FGF2 at 5 ng/ml failed to increase the growth of these cells (Fig. 3c) though inducing a slight acceleration of BrdU intake which was blocked by IMB-R1 (Additional file 1: Figure S2). Increasing doses of FGF2 did not change the outcome in MDAMB468 cells (data not shown).

IMB-R1 is polyclonal antisera, albeit with good specificity. Next, IMB-R1 was further antigen-affinity purified, and two middle fractions, IMB-R1A and IMB-R1B, examined for their effects on proliferation. At 8 μg/ml, IMB-R1B inhibited MG63 cell growth by 47 %, and IMB-R1A by 30 % (Fig. 3d), indicating that the adverse effect of IMB-R1 on cancer cells was in fact due to its anti-FGFR1 activity, and not any other, non-relevant component in the sera.

### IMB-R1 is more potent than other FGFR1-blocking agents

SU5402 and PD173074 are selective FGFR drug inhibitors, frequently (especially SU5402) utilized to block the tyrosine kinase activity of FGFRs [2]. The anti-FGFR1 antibody (clone # MAB765, R&D Systems) is one of the very few neutralizing antibodies against FGFR1 that have reached the market. The chemical inhibitors dose-dependently blocked the growth of both normal and cancer cells (Fig. 3e). The commercial FGFR1 antibody failed to reduce the basal growth of MG63 cells, even at doses up to 50 μg/ml (Fig. 3f).

### IMB-R1 induced cell apoptosis

To assess whether IMB-R1 could actively induce apoptosis, we next examined caspase 3 levels; it was induced by IMB-R1 (at 1:250 dilution) in MG63 cells starting from 10–16 h after treatment, reaching a maximum (2.7-fold) after 20 h (Fig. 4a, *left panel*). This apoptotic potency was then compared to that of staurosporine, which is also caspase-dependent [32]. Although staurosporine at 15 nM was sufficient to induce caspase 3 activity in MG63 cells, IMB-R1 at 1:250 was 3 times more potent (Fig. 4a, *middle and right panels*).

Further apoptotic analysis was performed using an Annexin V/PI dual staining approach in cells treated with IMB-R1 or SU5402, a widely used FGFR chemical inhibitor. Annexin V binds to phosphatidylserine, which only appears at the early stage of apoptosis, whilst PI can only stain cells at later stages of apoptosis. Therefore, positive staining for Annexin V indicates early apoptosis, whereas staining for both dyes suggests late apoptosis or cell death. Unstained cells were first acquired for gating purpose (data not shown). Both IMB-R1 and SU5402 treatment induced a significant shift in the cancer cell population from left (viable) to right (apoptotic) within the quadrant (Fig. 4b), indicating potent apoptotic activity. However, IMB-R1 was less toxic to normal osteoblasts and did not give rise to apoptosis in normal mammary epithelial cells (Additional file 1: Figure S3). Compared with the control, IMB-R1 gave rise to 2.6-fold more cell death in osteosarcoma cells (MG63), 2.7-fold more in breast cancer cells (MDAMB468 and T47D), 1.5-fold more in normal osteoblasts (hFOBs), with no cell death in normal mammary cells (MCF10A). Thus IMB-R1 appears more toxic to tumor cells than to healthy cells of the same tissue. In contrast, SU5402 induced apoptosis in both normal cells, being especially toxic for the MCF10A (~88-fold) (Additional file 1: Figure S3). SU5402 caused double the level of cell death in MG63, and ~5-8 times more in MDAMB468 and T47D cells. Therefore, at the dosages, SU5402 is less effective than IMB-R1 in killing osteosarcoma cells or certain breast cancer cells, and is more toxic than IMB-R1 to healthy mammary cells.

### IMB-R1 blocks FGFR1 signaling to downstream effectors

FGFR1 activation leads to the phosphorylation of an array of kinases or enzymes that mediate several signaling cascades [33–36]. FGF2 strongly activates two immediate effectors of FGFR1 in MG63 cells, FGFR substrate 2 (FRS2) and phospholipase Cγ (PLCγ), as well as the downstream extracellular signal-regulated kinases (ERK1/2) (Fig. 5a). In contrast, the activation of Akt or protein kinase C (PKC) was only minimal, confirming ERK is a major pathway for FGF2 signaling in these cells. ERK pathways control cell proliferation and survival, also in breast cancer cells exposed to FGF2 (Fig. 5a). However, in the presence of IMB-R1, the FGF2-induced activity of this kinase was greatly reduced (Fig. 5b).

U0126, a specific inhibitor of the upstream kinase of ERKs, was used to confirm the above results. The dose of U0126 required to completely shut down ERK signaling was approximately 25 μM (Fig. 5c). At this dose, U0126 induced significant cell death in all three cancer cell lines, with fold-induction being 13.4, 2.4 and 1.3 for the MG63, MDAMB468 and T47D cells respectively (Fig. 5d).

Next we studied the effect of IMB-R1 on proteins involved in apoptosis or tumor progression. IMB-R1 increased p53 levels in MG63, MCF10A and MDA-MB468 cells, and induced its phosphorylation in all cell types (Fig. 5e). The cell cycle inhibitor p21 is a target gene of p53. It was up-regulated by IMB-R1 in all cells tested. Retinoblastoma protein (Rb) was increased by IMB-R1 in hFOBs and MG63 cells. MDAMB468 cells did not express Rb in our hands, which is consistent with a previous report [37]. Thus Rb appeared to be a target molecule for IMB-R1 in bone tissue but not in the breast tissue. IMB-R1 also potently increased pro-apoptotic Bim protein levels in all the cells tested except the MDA-MB468. Notably, c-Myc, whose role in apoptosis has been established [38], was increased in all cells by IMB-R1.

### The global effect of IMB-R1 on the gene expression in cancer cells

**Fig. 6:**
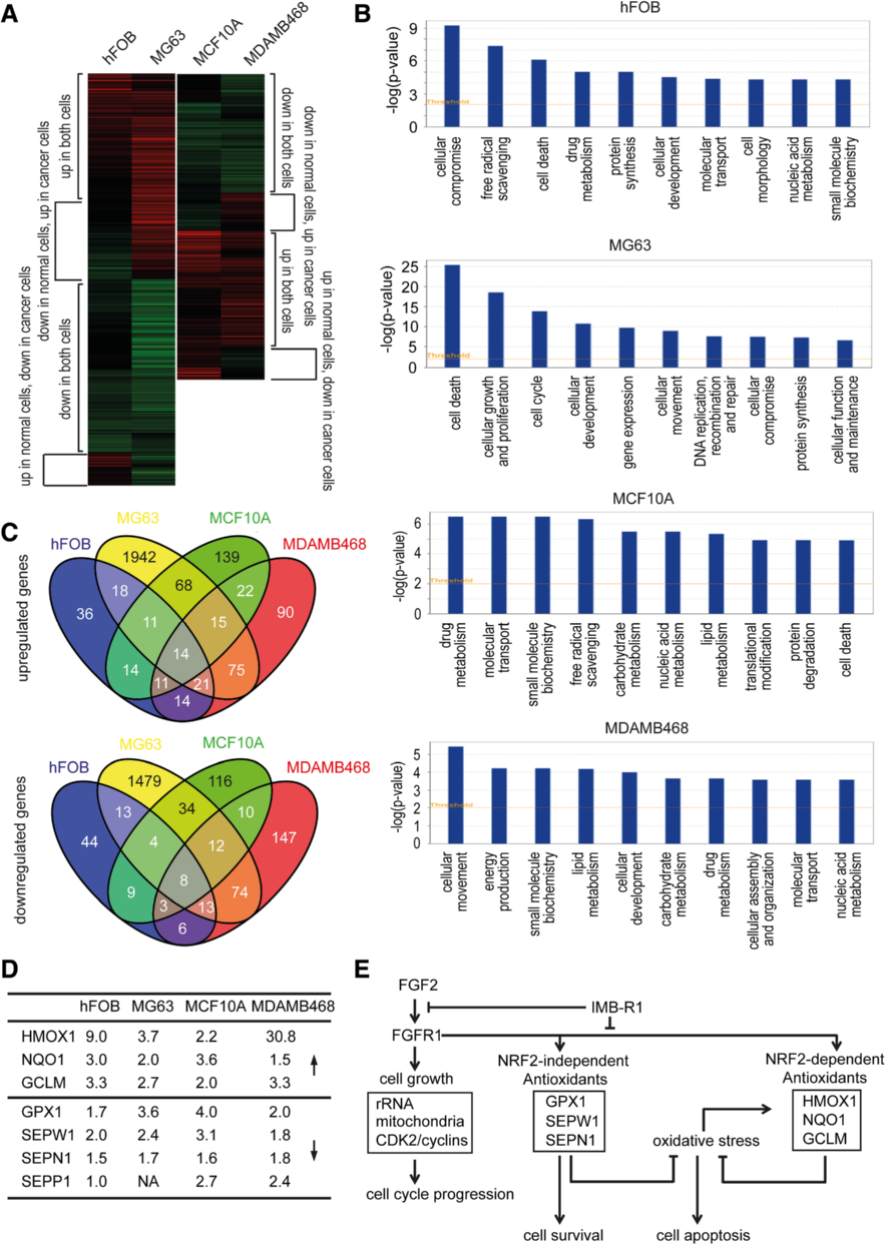
The effect of IMB-R1 on gene expression. Cells were treated with IMB-R1 (1:250 dilution) for 48 h and RNA extracted for microarray analysis. **a**, The heatmaps: Red, up-regulation; Green, down-regulation. **b**, Top 10 affected cellular functions by IMB-R1 as determined by Ingenuity Pathway Analysis. **c**, The number of common genes affected across the different cells. **d**, The antioxidant genes significantly regulated by IMB-R1. **e**, The proposed signaling mechanisms disrupted by IMB-R1 during FGF2/FGFR1 dependent cell growth and survival in cancer cells

To identify the mechanisms behind the apoptotic effect of IMB-R1, we performed gene profiling analysis; distinct profiles were revealed between normal cells and cancerous cells (Fig. 6a). With regard to the top 10 cellular functions affected by IMB-R1, “cell death” ranked as the first for MG63 cells and third for hFOBs, but last for the normal MCF10A and this gene ontology category was not evident for MDAMB468 cells (Fig. 6b). This again seems to confirm that bone tissue is more sensitive to IMB-R1 than breast tissue. Further analysis of the genes affected in common between different sets of cell groups revealed other pertinent gene lists (Fig. 6c, Additional file 1: Figure S4). The nuclear factor E2-related factor 2 (NRF2) target genes heme oxygenase (decycling) 1 (HMOX1), NAD(P)H:quinone oxidoreductase (NQO) 1 and glutamate-cysteine ligase (GCL) M were up-regulated whereas glutathione peroxidase (GPX) 1 and other selenoprotein genes (SEPW1, SEPN1 or SEPP1) were down-regulated in both cancer cells and normal cells (Fig. 6d, Additional file 1: Figure S4). These genes mostly appeared in the top 20 gene lists, suggesting NRF2- dependent and - independent antioxidative defense predominantly contributed to FGF/FGFR1-triggered cell survival (Fig. 6e), and served as the key mechanism driving IMB-R1 induced cell death.

### IMB-R1 recognizes FGFR1 in tumour biopsies

**Fig. 7:**
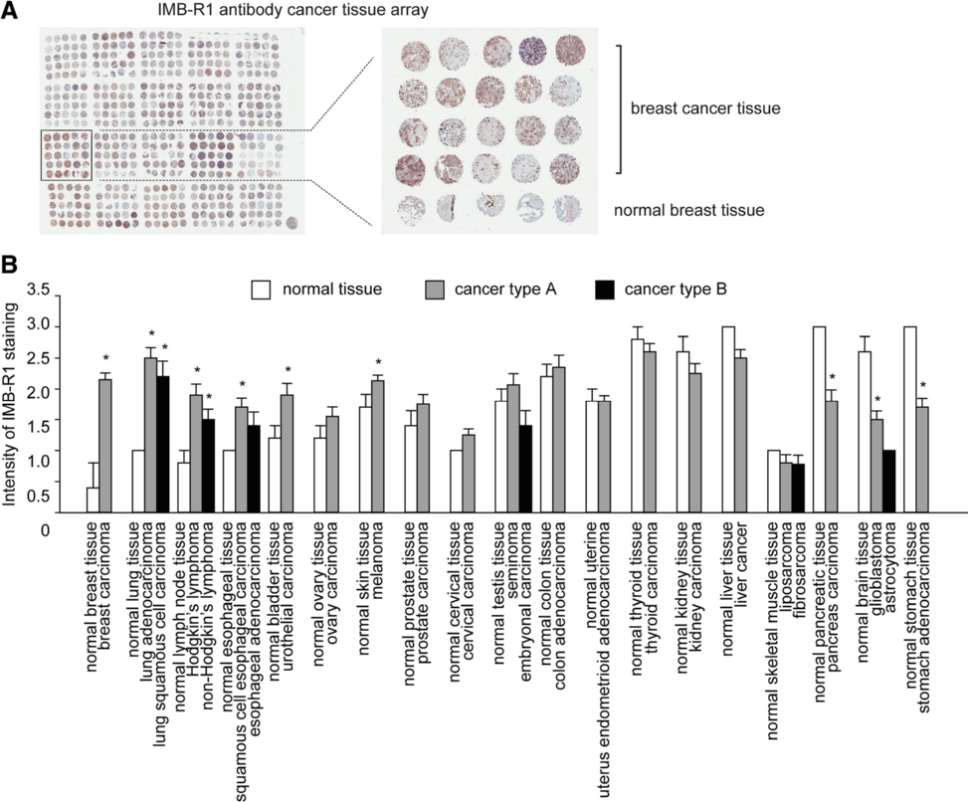
IMB-R1 targets FGFR1 in multiple human cancers. **a**, IMB-R1 histochemical staining from a human cancer tissue array. *Right panel*, enlarged image of boxed area highlighting increased FGFR1 expression (detected by IMB-R1) in breast cancer tissues from twenty separate donors compared with adjacent healthy breast tissue. **b**, The intensity of FGFR1 expression (detected by IMB-R1) was scored and the average scores for the various cancer tissues compared with those from adjacent healthy tissues

To determine the distribution of IMB-R1 in different cancers, IHC staining was performed on sections of nineteen separate human cancer tissues as well as the adjacent normal tissue. After staining, the DAB substrate yielded a dark brown end-product at the site of the target antigen, with hematoxylin providing the counterstaining of cell nuclei in blue (Fig. 7a). In most tissues, the location of IMB-R1 staining was in the cytoplasm. IMB-R1 staining was significantly stronger in cancer cells in the breast, lung, lymphoma, esophagus, bladder and ovary tissues as well as in melanoma cells compared to adjacent normal tissue, suggesting FGFR1 expression is increased in these tumors (Fig. 7b). Increased IMB-R1 staining was evident in invasive ductal carcinoma cells compared to gland epithelial cells in adjacent healthy tissue (Fig. 7a, *right panel*). This finding is consistent with a previous report that FGFR1 gene amplification was observed in breast, ovarian, lung, bladder and esophageal cancers [39]. Importantly, the greater binding of IMB-R1 in these tumor tissues than in the normal tissues indicated that IMB-R1 is able to target malignant cells in these tissues/organs relatively well over the adjacent normal cells.

## Discussion

The growth of many aggressive tumors involves FGF signaling which in turn is dependent on HS as an essential ‘co-receptor’. Here a highly specific FGFR1 antibody, IMB-R1, was engineered in an attempt to prevent the engagement of endogenous HS with FGFR1, so preventing formation of an active FGF-HS-FGFR1 signaling complex. The antibody recognizes specific epitopes that are adjacent to the unique heparin-binding domain within FGFR1, so disrupting the sugar-receptor association. Thus, our approach differs markedly from other strategies that have sought to block ligand-receptor interactions by concentrating on the ligand-binding site. Our approach not only yields a novel agent for preclinical testing, but a novel way of blocking all HS-dependent interactions involved in carcinogenesis.

Characterization of the mechanism of action of IMB-R1 demonstrated that it selectively affects cancer cell survival by preventing the formation of FGF/FGFR1 complexes, thereby inhibiting the FGF/FGFR1 signaling axis. This is particularly important because heparin is known to facilitate FGF and FGFR1 dimerization [40], which has resulted in attempts to modulate heparin’s action through competition with other carbohydrate compounds [41–44]. Rather than compete against heparin activity, here we sought to block heparin binding and subsequent ligand/receptor dimerization by using an antibody approach.

FGFR1 is one of the most widely expressed FGFRs [11], and here we confirmed that FGFR1 is strongly expressed in both normal and cancer tissues. We note that treatment with IMB-R1 may result in altered levels of FGF-R1 expressed on the cell surface by interfering with FGF-2/FGFR-1 binding and subsequent receptor endocytosis (internalisation) [45]. FGFR1 mRNA and/or protein levels are both significantly elevated in distinct cancers (e.g., breast and bone cancer), suggesting that the FGFR1 receptor is a rational target for therapeutic intervention. We found that normal mammary epithelial cells are less sensitive to IMB-R1 than breast cancer cells, which in turn suggests an antibody-based regime for preferential elimination of breast cancer cells with minimal harm towards normal mammary cells at the appropriate dosage is possible. However, this needs to be balanced against the finding that normal osteoblastic cells are more sensitive to IMB-R1 than osteosarcoma cells. Thus, the antibody acts in a cell and tissue type -specific manner, presumably due to differences in the relative expression of FGFR1. The latter result, obtained with cultured cells, provides a counter-indication for its utility *in vivo*. Systemic administration of the IMB-R1 antibody might exacerbate the osteolysis induced by metastatic breast cancers. On the other hand, local delivery of the antibody into mammary gland tumors might prove effective in constraining growth and thus offer a less invasive therapy for inhibition of the primary breast tumor mass.

We have used a variety of methods to determine the mechanism by which the IMB-R1 antibody affects cell survival. The cDNA microarray expression analysis indicates that IMB-R1 consistently down-regulates the expression of proteins that contain selenocysteine (GPX1, SEPP and SEPW), which are an important component of antioxidant defense in human cells [46]. In view of their protective role for tumor cells against oxidative insult, and their overexpression in many cancer cells, selenoproteins have recently become targets for anti-cancer therapies [47]. Suppressing selenoprotein expression leads to increased oxidative stress in tumor cells and subsequent apoptosis, which may also contribute to the observed cancer cell growth arrest and death induced by IMB-R1. Collectively, our data suggest that suppression of antioxidants to induce apoptosis and inhibition of FGFR1-dependent mitogenic activity may together represent two principal mechanisms for the anti-oncogenic effects of the IMB-R1 antibody.

As well as inactivation of selenoproteins, the data also reveal that IMB-R1 increases the expression of the HMOX1, NQO1 and GCLM. These and other genes are downstream targets of the transcription factor NRF2, which is a master transcriptional regulator of the oxidative stress response. In normal cells, NRF2-mediated mitigation of oxidative stress prevents cancer initiation and progression by removing excessive ROS, so preventing DNA damage and avoiding spontaneous mutations [48, 49]. However, one caveat for these beneficial cytoprotective effects is that overactivation of NRF2 can be oncogenic, through the survival of cancer cells that have accumulated oxidative damage and genetic mutations [50, 51]. Importantly, FGF signaling is known to activate NRF2 to provide cytoprotection against oxidative stress [52]. Therefore, if our hypothesis is correct, IMB-R1 should decrease the expression of NRF2-dependent genes.

To explain the paradoxical effects of IMB-R1 on selenoproteins and NRF2 target proteins, we suggest that the opposing expression profiles for the two classes of anti-oxidant proteins may reflect regulatory feedback. The IMB-R1 antibody might initially mediate suppression of selenoproteins, triggering oxidative stress, which in turn might induce NRF2 and activate the program of NRF2 - responsive genes (HMOX1, NQO1 and GCLM). It appears that the reduction in selenoprotein expression prevails, so inducing apoptosis upon IMB-R1 treatment. Hence our data indicates that a key survival function of FGFR1 is to support synthesis of selenoproteins. The universal alteration of different groups of antioxidant genes in opposing directions further indicate that IMB-R1- induced cell death is a net outcome of unbalanced oxidative insult *vs* antioxidative defense.

In specifically blocking signaling of FGF2/HS complexes through FGFR1, IMB-R1 selectively affects cancer cell survival and exhibits reduced non-specific toxicity compared to chemical pathway inhibitors. This set of attributes compares favorably with those of other FGFR inhibitors, including SU5402 [53] and PD173074 [54], both of which tend to be indiscriminately toxic to both normal and cancer cells. The efficacy of IMB-R1 also compares favorably to the commercial neutralizing FGFR1 antibody, MAB765 that failed to reduce the basal growth of cancer cells. One limitation of this particular antibody is that it is directed against the FGFR1 IIIb isoform, which is preferentially expressed in epithelial cells. However, MAB765 does not antagonize the activity of the IIIc isoform, the form which is expressed prominently in mesenchymal cells. In contrast, IMB-R1 recognizes both isoforms, so offering inhibition of FGFR1 signaling in cancers of either epithelial or mesenchymal origin. IMB-R1 differs from other existing FGFR1-neutralizing antibodies in that it expressly disrupts HS-FGFR1 interactions, highlighting the importance of targeting heparin-binding sites as a potential anti-cancer strategy.

## Conclusions

IMB-R1 differs from other existing FGFR1-neutralizing antibodies in that it expressly disrupts HS-FGFR1 interactions, highlighting the importance of targeting heparin-binding sites as a potential anti-cancer strategy, not just for FGFRs but for any cancer related heparin-binding proteins.

## Methods

### Chemicals and inhibitors

SU5402, Staurosporine and U0126 were obtained from Merck. PD173074, protease inhibitor cocktails and other chemicals were purchased from Sigma-Aldrich.

### Cell culture

Cells were purchased from ATCC and maintained in the corresponding recommended medium, except human osteosarcoma cells (OS1) [55] that were cultured in DMEM (1000 mg/L glucose) supplemented with 10 % FCS, 2 mM L-glutamine, 25 mM HEPES (Biopolis Shared Facility, A*STAR, Singapore) and antibiotics. Media changes were performed every 2–3 days.

### Taqman real-time quantitative PCR analysis

Cells were grown in triplicates and treated as indicated. The mRNA expression of target genes were analysed using the Taqman® real-time PCR method as described previously [56]. Primers and probes were all pre-designed by Applied Biosystems.

### Western blot analysis

Cells were treated as indicated and lysed in Laemmli buffer at 95 °C for 5 min. The denatured protein lysates (~20 μl) were separated by sodium dodecyl sulfate polyacrylamide gel electrophoresis and proteins transferred to nitrocellulose membranes. The blots were divided into three to five horizontal strips guided by protein standards stained by Ponceau Red to permit analysis of multiple proteins from the same sample without antibody stripping. Thereafter membranes were immunoblotted, protein targets visualized and their levels quantified as described previously [56]. The p21 antibody was obtained from BD Biosciences. The antibodies against FGFRs or p53 were purchased from Santa Cruz. FGFR1 antibody (#MAB765) was from R&D Systems. All other antibodies were supplied by Cell Signaling Technology.

### Antibody engineering

The peptide SSSEEKETDNTKPNR, located immediately upstream of the heparin-binding domain of FGFR1, was chosen as the antigen for the production of rabbit polyclonal FGFR1-neutralising antibodies as described previously [56]. The rabbit antiserum was designated as IMB-R1, and was further affinity-purified using Reacti-Gel beads (Thermo Scientific) coupled with the above peptide. With this method we obtained two purified polyclonal antibodies, IMB-R1A and IMB-R1B, from two rabbit sera.

### Sandwich Enzyme-linked immunosorbent assay (ELISA)

Maxisorp™ EIA plates (Thermo Scientific) were coated with 0.5 μg/ml goat anti-human IgG-Fc (Jackson ImmunoResearch Laboratories) in PBS at 4 °C overnight. Thereafter the plate was blocked with 2 % bovine serum albumin (BSA) for 1 h. Recombinant human FGFRs-Fc (R&D systems) (500 ng/ml) or the control human IgG-Fc (Abcam) was then added for 2 h, followed by incubation with IMB-R1 or the control rabbit IgG (Invitrogen) at the indicated doses for 1 h. The bound antibodies were detected with 0.5 μg/ml HRP-conjugated goat anti-rabbit IgG (Jackson ImmunoResearch Laboratories) and visualized with TMB substrate (Thermo Scientific). The color was read at 450 nm using the Victor^3^ multilabel plate reader (PerkinElmer). All reactions were performed at room temperature, unless indicated otherwise, and protected from light, with each step followed by extensive washing in blocking buffer. The readings were normalized against the controls.

### Protein-GAG binding assay

To investigate whether IMB-R1 prevented heparin/HS binding to FGFR1, 50 μg/ml heparin was coated onto GAG-binding plates in the standard assay buffer provided (Iduron). After blocked with 0.5 % BSA, the plate was incubated with 2 μg/ml FGFR-Fc in PBS for 2 h at 37 °C. FGFR-Fc bound to heparin was then detected using 0.5–1 μg/ml HRP-conjugated goat anti-human IgG-Fc (Jackson ImmunoResearch Laboratories) for 1 h and visualized as described above.

To examine whether IMB-R1 affected the interaction between FGFRs and FGF2, 5 μg/ml heparin was coated onto the plate (using the standard assay buffer) as a substrate to bind FGF2. The heparin-coated surface was blocked with 0.5 % fish gelatin before 200 ng/ml FGF2 (R&D systems) in PBS was added for 2 h at 37 °C. Next, FGFR-Fc (500 ng/ml) was pre-complexed in PBS with increasing amounts of IMB-R1 (on ice for 2 h). The complex was then applied to the FGF2 surface described above for 1.5 h. The amount of FGFR-Fc bound to FGF2 was determined as described above.

### Receptor Tyrosine Kinase array

The phosphorylation status of the FGFRs was determined with the Human Phospho-Receptor Tyrosine Kinase Array (R&D Systems). MG63 cells were seeded at 20,000 cells/cm^2^ for 1 day and deprived of serum for 48 h before treated with 20 ng/ml FGF2 in the presence of IMB-R1 or rabbit IgG (at 1:250 dilution) for 5 min. The cells were then rinsed with PBS and lysed with the buffer provided in the kit. Cell lysates (250 μg) were assayed for of FGFR kinase activity following the manufacturer’s instruction.

### Proliferation assay

The cells were plated in triplicate at 20,000 cells/cm^2^ except for the MDAMB468 cells, that were at 100,000 cells/cm^2^. They were treated with indicated doses of IMB-R1 or other reagents for 2 days, before viable cell numbers were assessed by the GUAVA Flow Cytometry Viacount Program as described previously [56].

### Annexin V- propidium iodide (PI) staining

Cells were plated at the densities nominated above and allowed to adhere overnight. Cells were treated with reagents for 24 h and then stained with Annexin V-FITC and/or PI [46] protected from light for 15 min.. Fluorescent cells were detected by BD FACS Array (BD Biosciences), and viable and apoptotic cells analyzed using FlowJo software (Tree Star Inc). The unstained cells were used for gating purposes.

### Caspase 3 activity assay

MG63 cells were seeded at 20,000 cells/cm^2^ and treated as indicated. Cells were then lysed and Caspase 3 activity measured using the Caspase-3 Colorimetric Assay Kit (BioVision) following the manufacturer’s instructions.

### Microarray

Three consecutive passages of cells were treated as indicated and the total RNA extracted in TRIzol®-reagent and purified with the PureLink™ RNA mini kit (Invitrogen). The purified RNA was processed to cRNA using the Illumina® TotalPrep™-96 RNA Amplification kit (Ambion) as per the manufacturer’s instructions. cRNA was hybridized to the probes on the Human HT-12 v4 Expression BeadChip (Illumina). After washing and staining, the chip was scanned by the BeadArray™ Reader. The data was processed and heatmaps were generated using GenomeStudio software. The gene expression data were further analysed using GeneSpring GC 11.0 software and DAVID Bioinformatics Resources 6.7 (http://david.abcc.ncifcrf.gov) [57]. The function analysis was performed using Ingenuity Pathways Analysis software. The overlapping of gene targets in different cells was analysed using VENNY software (http://bioinfogp.cnb.csic.es/tools/venny/index.html).

### Cancer tissue array

The human multiple organ cancer tissue array (US Biomax, Inc) contains 19 types of cancer with 20 cases/type and 5 cases/type of normal controls. The sections were stained with IMB-R1 using a standard immunohistochemistry (IHC) paraffin staining method. The section was first deparaffinized and heat-induced antigen retrieval performed. After blocking in horse serum, the section was incubated with IMB-R1 or rabbit serum at a dilution of 1:800 for 1 h. The bound IMB-R1 was detected with ImmPRESS™ peroxidase anti-rabbit (Vector Laboratories) for 30 min followed by addition of peroxidase substrate DAB solution (DAKO Cytomation). Thereafter the section was counterstained with Hematoxylin QS (Vector Labs) and mounted in permanent mounting medium (Sigma). All steps were performed at room temperature, and between incubations sections were rinsed free of non-specific binding. The total positive cell numbers and intensity of the antibody staining were measured by ImageScope (Aperio Scanning System) and 20x object images captured. The intensity of staining was scored as negative (0), weak (1+), moderate (2+), or strong (3+).

### Statistical analysis

Each experiment was repeated at least three times and numeric data were expressed as mean ± SD of triplicate samples. Differences among treatments were analyzed by Student’s *t* test. Significant differences were considered as those with a *p* value < 0.05.

## Additional file

Additional file 1:
**Supplementary data. Figure S1.** A, Proposed interaction between IMB-R1 and FGFR1. B, Affinity of FGFR to heparin in the presence or absence of IMB-R1 was presented as in Fig. 2b. The percentage reduction of FGFR binding to heparin as determined by a comparison of means was further calculated and shown as in the bar graphs. C, Affinity of FGFR to FGF2 in the presence or absence of IMB-R1 was shown in Fig. 2c. Here the percentage reduction of FGFR binding to FGF2 as determined by a comparison of means was plotted as bar graphs. **Figure S2.** The effect of IMB-R1 on the proliferation rate of cancer cells were determined using 5-Bromo-2′- deoxyuridine (BrdU) incorporation assay. Cells were plated at 20,000 cells/cm^2^ except MDAMB468 which were seeded at 100,000 cells/cm^2^ and allowed to adhere overnight. Cell cycling was then arrested by serum deprivation for 48 h. The quiescent cells were then treated with IMB-R1 or vehicle for 1 h followed by FGF2 treatment (20 ng/ml for MG63, 5 ng/ml for MDAMB468 and T47D) for another 24 h. Cells were subsequently labeled with BrdU (Roche) for 3 h. The incorporated BrdU was detected as per manufacturer’s instructions and the absorbance was measured at 370 nm. **Figure S3.** The apoptotic effect of IMB-R1 and SU5402 on normal osteoblast cells and normal mammary gland cells (hFOB and MCF10A, respectively) were assessed as described in Fig. 4b. **Figure S4.** The further analysis of microarray data described in Fig. 6. Genes affected in all 4 cells, and the Top 20 common genes affected in various groups were indicated.

## Abbreviations

BSA: Bovine serum albumin
BrdU: 5-Bromo-2′- deoxyuridine
DMEM: Dulbecco's Modified Eagle Medium
ECL: enhanced chemiluminescence
ECM: extracellular matrix
ELISA: Enzyme-linked immunosorbent assay
ERK: extracellular signal-regulated kinase
FCS: fetal calf serum
FGF: fibroblast growth factor
FGFR: fibroblast growth factor receptor
FRS2: fibroblast growth factor receptor substrate 2
GAG: glycosaminoglycan
HBD: heparin-binding domain
HRP: horseradish-peroxidase
HS: heparan sulfate
IC50: the half maximal inhibitory concentration
IHC: immunohistochemistry
MEK: MAP kinase kinase
PDGFR: platelet-derived growth factor receptor
PLCγ: phospholipase Cγ
PKC: protein kinase C
PI: propidium iodide
Rb: Retinoblastoma protein
RTK: receptor tyrosine kinase
TKI: tyrosine kinase inhibitor
VEGFR: vascular endothelial growth factor receptor

## References

[1] Jeffers M, LaRochelle WJ, Lichenstein HS (2002). Fibroblast growth factors in cancer: therapeutic possibilities. Expert Opin Ther Targets.

[2] Turner N, Grose R (2010). Fibroblast growth factor signalling: from development to cancer. Nat Rev Cancer.

[3] Dienstmann R, Rodon J, Prat A, Perez-Garcia J, Adamo B, Felip E (2014). Genomic aberrations in the FGFR pathway: opportunities for targeted therapies in solid tumors. Ann Oncol.

[4] Fearon AE, Gould CR, Grose RP (2013). FGFR signalling in women's cancers. Int J Biochem Cell Biol.

[5] Greenman C, Stephens P, Smith R, Dalgliesh GL, Hunter C, Bignell G (2007). Patterns of somatic mutation in human cancer genomes. Nature.

[6] Ho HK, Yeo AH, Kang TS, Chua BT (2014). Current strategies for inhibiting FGFR activities in clinical applications: opportunities, challenges and toxicological considerations. Drug Discov Today.

[7] Qing J, Du X, Chen Y, Chan P, Li H, Wu P (2009). Antibody-based targeting of FGFR3 in bladder carcinoma and t(4;14)-positive multiple myeloma in mice. J Clin Invest.

[8] Zhao WM, Wang L, Park H, Chhim S, Tanphanich M, Yashiro M (2010). Monoclonal antibodies to fibroblast growth factor receptor 2 effectively inhibit growth of gastric tumor xenografts. Clin Cancer Res.

[9] Karajannis MA, Vincent L, Direnzo R, Shmelkov SV, Zhang F, Feldman EJ (2006). Activation of FGFR1beta signaling pathway promotes survival, migration and resistance to chemotherapy in acute myeloid leukemia cells. Leukemia.

[10] Johnson DE, Williams LT (1993). Structural and functional diversity in the FGF receptor multigene family. Adv Cancer Res.

[11] Hughes SE (1997). Differential expression of the fibroblast growth factor receptor (FGFR) multigene family in normal human adult tissues. J Histochem Cytochem.

[12] Ornitz DM, Itoh N (2001). Fibroblast growth factors. Genome Biol.

[13] Kan M, Wang F, Xu J, Crabb JW, Hou J, McKeehan WL (1993). An essential heparin-binding domain in the fibroblast growth factor receptor kinase. Science.

[14] Plotnikov AN, Schlessinger J, Hubbard SR, Mohammadi M (1999). Structural basis for FGF receptor dimerization and activation. Cell.

[15] Friedl A, Chang Z, Tierney A, Rapraeger AC (1997). Differential binding of fibroblast growth factor-2 and −7 to basement membrane heparan sulfate: comparison of normal and abnormal human tissues. Am J Pathol.

[16] Nurcombe V, Ford MD, Wildschut JA, Bartlett PF (1993). Developmental regulation of neural response to FGF-1 and FGF-2 by heparan sulfate proteoglycan. Science.

[17] Rapraeger AC, Krufka A, Olwin BB (1991). Requirement of heparan sulfate for bFGF-mediated fibroblast growth and myoblast differentiation. Science.

[18] Yayon A, Klagsbrun M, Esko JD, Leder P, Ornitz DM (1991). Cell surface, heparin-like molecules are required for binding of basic fibroblast growth factor to its high affinity receptor. Cell.

[19] Furdui CM, Lew ED, Schlessinger J, Anderson KS (2006). Autophosphorylation of FGFR1 kinase is mediated by a sequential and precisely ordered reaction. Mol Cell.

[20] Greulich H, Pollock PM (2011). Targeting mutant fibroblast growth factor receptors in cancer. Trends Mol Med.

[21] Freier K, Schwaenen C, Sticht C, Flechtenmacher C, Muhling J, Hofele C (2007). Recurrent FGFR1 amplification and high FGFR1 protein expression in oral squamous cell carcinoma (OSCC). Oral Oncol.

[22] Cihoric N, Savic S, Schneider S, Ackermann I, Bichsel-Naef M, Schmid RA (2014). Prognostic role of FGFR1 amplification in early-stage non-small cell lung cancer. Br J Cancer.

[23] Turner N, Pearson A, Sharpe R, Lambros M, Geyer F, Lopez-Garcia MA (2010). FGFR1 amplification drives endocrine therapy resistance and is a therapeutic target in breast cancer. Cancer Res.

[24] Courjal F, Cuny M, Simony-Lafontaine J, Louason G, Speiser P, Zeillinger R (1997). Mapping of DNA amplifications at 15 chromosomal localizations in 1875 breast tumors: definition of phenotypic groups. Cancer Res.

[25] Murphy T, Darby S, Mathers ME, Gnanapragasam VJ (2010). Evidence for distinct alterations in the FGF axis in prostate cancer progression to an aggressive clinical phenotype. J Pathol.

[26] Gorringe KL, Jacobs S, Thompson ER, Sridhar A, Qiu W, Choong DY (2007). High-resolution single nucleotide polymorphism array analysis of epithelial ovarian cancer reveals numerous microdeletions and amplifications. Clin Cancer Res.

[27] Simon R, Richter J, Wagner U, Fijan A, Bruderer J, Schmid U (2001). High-throughput tissue microarray analysis of 3p25 (RAF1) and 8p12 (FGFR1) copy number alterations in urinary bladder cancer. Cancer Res.

[28] Knights V, Cook SJ (2010). De-regulated FGF receptors as therapeutic targets in cancer. Pharmacol Ther.

[29] Ling L, Murali S, Dombrowski C, Haupt LM, Stein GS, van Wijnen AJ (2006). Sulfated glycosaminoglycans mediate the effects of FGF2 on the osteogenic potential of rat calvarial osteoprogenitor cells. J Cell Physiol.

[30] Hanneken A (2001). Structural characterization of the circulating soluble FGF receptors reveals multiple isoforms generated by secretion and ectodomain shedding. FEBS Lett.

[31] Dombrowski C, Helledie T, Ling L, Grunert M, Canning CA, Jones CM (2013). FGFR1 signaling stimulates proliferation of human mesenchymal stem cells by inhibiting the cyclin-dependent kinase inhibitors p21(Waf1) and p27(Kip1). Stem Cells.

[32] Belmokhtar CA, Hillion J, Segal-Bendirdjian E (2001). Staurosporine induces apoptosis through both caspase-dependent and caspase-independent mechanisms. Oncogene.

[33] Altomare DA, Testa JR (2005). Perturbations of the AKT signaling pathway in human cancer. Oncogene.

[34] Eswarakumar VP, Lax I, Schlessinger J (2005). Cellular signaling by fibroblast growth factor receptors. Cytokine Growth Factor Rev.

[35] Klint P, Claesson-Welsh L (1999). Signal transduction by fibroblast growth factor receptors. Front Biosci.

[36] Peters KG, Marie J, Wilson E, Ives HE, Escobedo J, Del Rosario M (1992). Point mutation of an FGF receptor abolishes phosphatidylinositol turnover and Ca2+ flux but not mitogenesis. Nature.

[37] Lee EY, To H, Shew JY, Bookstein R, Scully P, Lee WH (1988). Inactivation of the retinoblastoma susceptibility gene in human breast cancers. Science.

[38] Prendergast GC (1999). Mechanisms of apoptosis by c-Myc. Oncogene.

[39] Daniele G, Corral J, Molife LR, de Bono JS (2012). FGF receptor inhibitors: role in cancer therapy. Curr Oncol Rep.

[40] Miao HQ, Ornitz DM, Aingorn E, Ben-Sasson SA, Vlodavsky I (1997). Modulation of fibroblast growth factor-2 receptor binding, dimerization, signaling, and angiogenic activity by a synthetic heparin-mimicking polyanionic compound. J Clin Invest.

[41] Gagliardi A, Hadd H, Collins DC (1992). Inhibition of angiogenesis by suramin. Cancer Res.

[42] Zugmaier G, Lippman ME, Wellstein A (1992). Inhibition by pentosan polysulfate (PPS) of heparin-binding growth factors released from tumor cells and blockage by PPS of tumor growth in animals. J Natl Cancer Inst.

[43] Szabo S, Vattay P, Scarbrough E, Folkman J (1991). Role of vascular factors, including angiogenesis, in the mechanisms of action of sucralfate. Am J Med.

[44] Miao HQ, Ishai-Michaeli R, Peretz T, Vlodavsky I (1995). Laminarin sulfate mimics the effects of heparin on smooth muscle cell proliferation and basic fibroblast growth factor-receptor binding and mitogenic activity. J Cell Physiol.

[45] Sandilands E, Akbarzadeh S, Vecchione A, McEwan DG, Frame MC, Heath JK (2007). Src kinase modulates the activation, transport and signalling dynamics of fibroblast growth factor receptors. EMBO Rep.

[46] Tapiero H, Townsend DM, Tew KD (2003). The antioxidant role of selenium and seleno-compounds. Biomed Pharmacother.

[47] Arner ES, Holmgren A (2006). The thioredoxin system in cancer. Semin Cancer Biol.

[48] Klaunig JE, Kamendulis LM, Hocevar BA (2010). Oxidative stress and oxidative damage in carcinogenesis. Toxicol Pathol.

[49] Tudek B, Winczura A, Janik J, Siomek A, Foksinski M, Olinski R (2010). Involvement of oxidatively damaged DNA and repair in cancer development and aging. Am J Transl Res.

[50] Homma S, Ishii Y, Morishima Y, Yamadori T, Matsuno Y, Haraguchi N (2009). Nrf2 enhances cell proliferation and resistance to anticancer drugs in human lung cancer. Clin Cancer Res.

[51] Kensler TW, Wakabayashi N (2010). Nrf2: friend or foe for chemoprevention?. Carcinogenesis.

[52] Vargas MR, Pehar M, Cassina P, Martinez-Palma L, Thompson JA, Beckman JS (2005). Fibroblast growth factor-1 induces heme oxygenase-1 via nuclear factor erythroid 2-related factor 2 (Nrf2) in spinal cord astrocytes: consequences for motor neuron survival. J Biol Chem.

[53] Mohammadi M, McMahon G, Sun L, Tang C, Hirth P, Yeh BK (1997). Structures of the tyrosine kinase domain of fibroblast growth factor receptor in complex with inhibitors. Science.

[54] Skaper SD, Kee WJ, Facci L, Macdonald G, Doherty P, Walsh FS (2000). The FGFR1 inhibitor PD 173074 selectively and potently antagonizes FGF-2 neurotrophic and neurotropic effects. J Neurochem.

[55] Pereira BP, Zhou Y, Gupta A, Leong DT, Aung KZ, Ling L (2009). Runx2, p53, and pRB status as diagnostic parameters for deregulation of osteoblast growth and differentiation in a new pre-chemotherapeutic osteosarcoma cell line (OS1). J Cell Physiol.

[56] Ling L, Dombrowski C, Foong KM, Haupt LM, Stein GS, Nurcombe V (2010). Synergism between Wnt3a and heparin enhances osteogenesis via a phosphoinositide 3-kinase/Akt/RUNX2 pathway. J Biol Chem.

[57] da Huang W, Sherman BT, Lempicki RA (2009). Bioinformatics enrichment tools: paths toward the comprehensive functional analysis of large gene lists. Nucleic Acids Res.

