# Supplementary material for: Targeting the heparin-binding domain of fibroblast growth factor receptor 1 as a potential cancer therapy
Source: Mol Cancer. 2015 Jul 23;14:136. doi: 10.1186/s12943-015-0391-4 (PMC4511971; doi:10.1186/s12943-015-0391-4)
Supplement: Additional file 1: — Supplementary data. Figure S1. A, Proposed interaction between IMB-R1 and FGFR1. B, Affinity of FGFR to heparin in the presence or absence of IMB-R1 was presented as in Fig. 2b. The percentage reduction of FGFR binding to heparin as determined by a comparison of means was further calculated and shown as in the bar graphs. C, Affinity of FGFR to FGF2 in the presence or absence of IMB-R1 was shown in Fig. 2c. Here the percentage reduction of FGFR binding to FGF2 as determined by a comparison of means was plotted as bar graphs. Figure S2. The effect of IMB-R1 on the proliferation rate of cancer cells were determined using 5-Bromo-2′- deoxyuridine (BrdU) incorporation assay. Cells were plated at 20,000 cells/cm2 except MDAMB468 which were seeded at 100,000 cells/cm2 and allowed to adhere overnight. Cell cycling was then arrested by serum deprivation for 48 h. The quiescent cells were then treated with IMB-R1 or vehicle for 1 h followed by FGF2 treatment (20 ng/ml for MG63, 5 ng/ml for MDAMB468 and T47D) for another 24 h. Cells were subsequently labeled with BrdU (Roche) for 3 h. The incorporated BrdU was detected as per manufacturer’s instructions and the absorbance was measured at 370 nm. Figure S3. The apoptotic effect of IMB-R1 and SU5402 on normal osteoblast cells and normal mammary gland cells (hFOB and MCF10A, respectively) were assessed as described in Fig. 4b. Figure S4. The further analysis of microarray data described in Fig. 6. Genes affected in all 4 cells, and the Top 20 common genes affected in various groups were indicated. [file 12943_2015_391_MOESM1_ESM.pdf]

Supplementary Data:

Figure Legends

Supplementary Fig.1. A, Proposed interaction between IMB-R1 and FGFR1. B, Affinity of FGFR to heparin in the presence or absence of IMB-R1 was presented as in Fig. 2B. The percentage reduction of FGFR binding to heparin as determined by a comparison of means was further calculated and shown as in the bar graphs. C, Affinity of FGFR to FGF2 in the presence or absence of IMB-R1 was shown in Fig. 2C. Here the percentage reduction of FGFR binding to FGF2 as determined by a comparison of means was plotted as bar graphs.

Supplementary Fig.2. The effect of IMB-R1 on the proliferation rate of cancer cells were determined using 5-Bromo-2′- deoxyuridine (BrdU) incorporation assay. Cells were plated at 20,000 cells/cm^2 except MDAMB468 which were seeded at 100,000 cells/cm^2 and allowed to adhere overnight. Cell cycling was then arrested by serum deprivation for 48 h. The quiescent cells were then treated with IMB-R1 or vehicle for 1 h followed by FGF2 treatment (20 ng/ml for MG63, 5 ng/ml for MDAMB468 and T47D) for another 24 h. Cells were subsequently labeled with BrdU (Roche) for 3 h. The incorporated BrdU was detected as per manufacturer's instructions and the absorbance was measured at 370 nm.

Supplementary Fig.3. The apoptotic effect of IMB-R1 and SU5402 on normal osteoblast cells and normal mammary gland cells (hFOB and MCF10A, respectively) were assessed as described in Fig. 4B.

Supplementary Fig.4. The further analysis of microarray data described in Fig. 6. Genes affected in all 4 cells, and the Top 20 common genes affected in various groups were indicated.

Supplementary Fig. 1

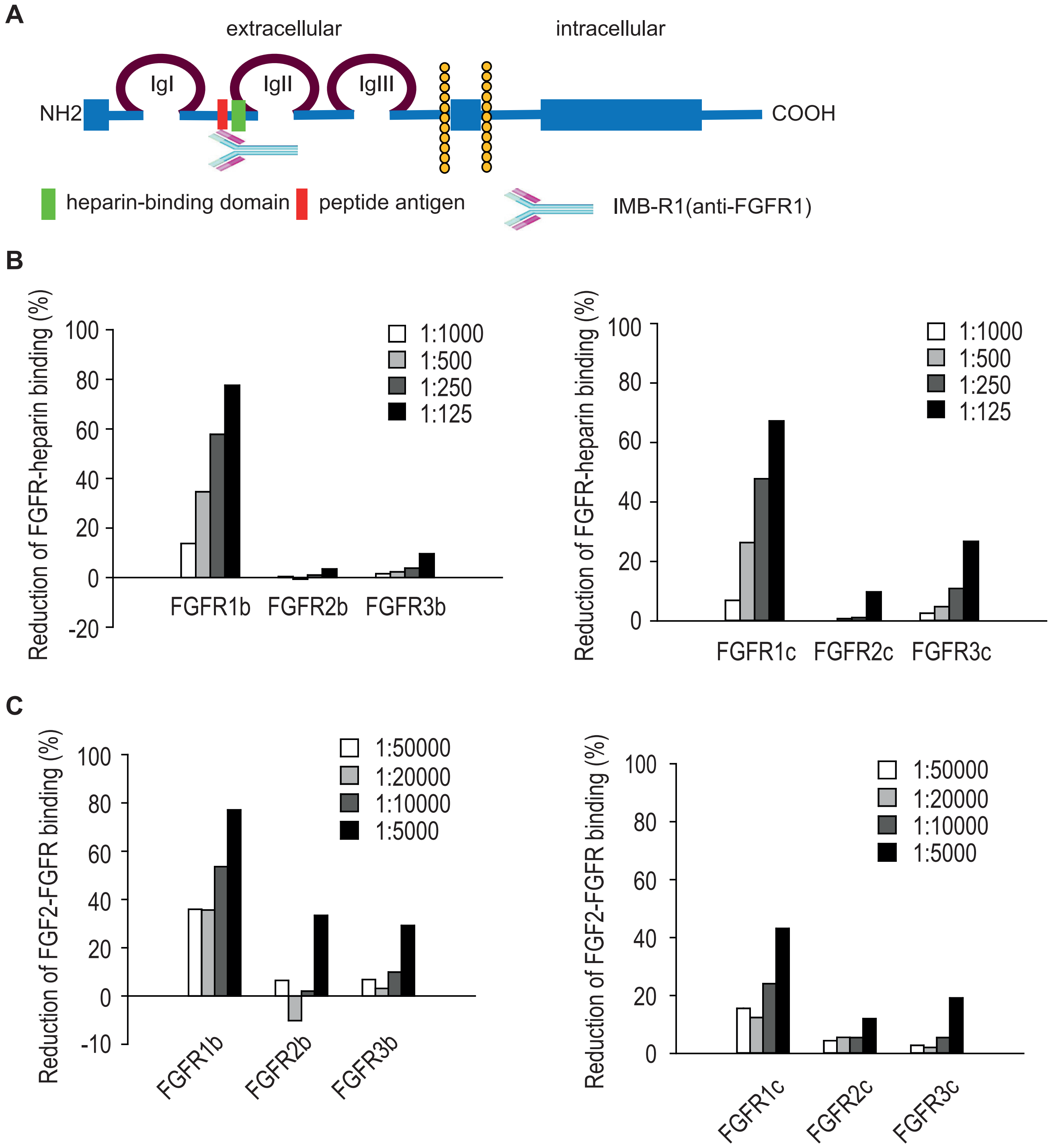

Supplementary Fig. 2

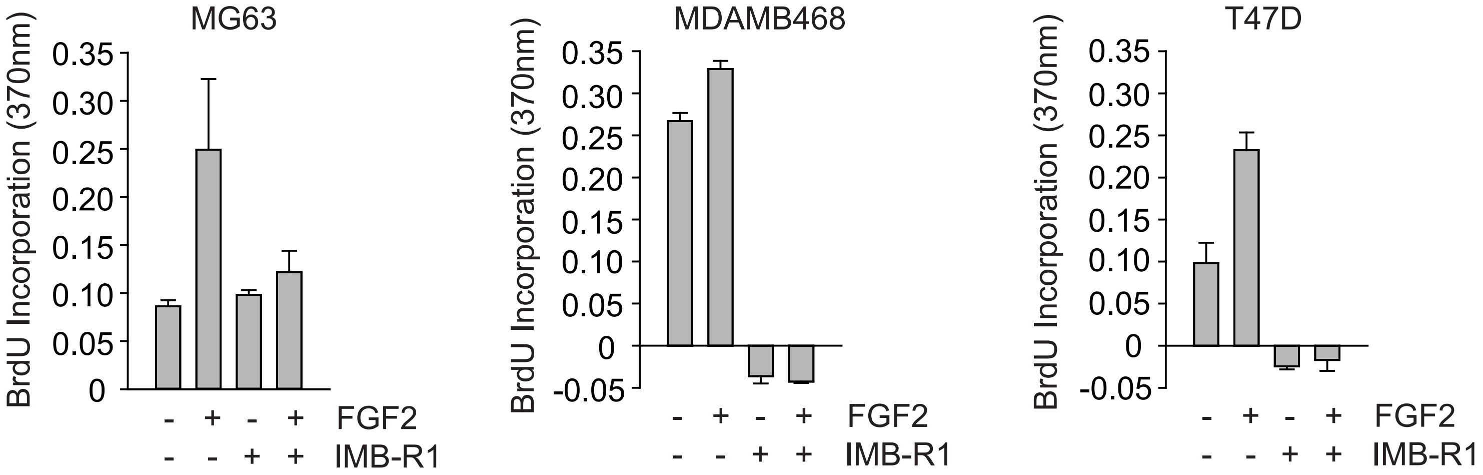

Supplementary Fig. 3

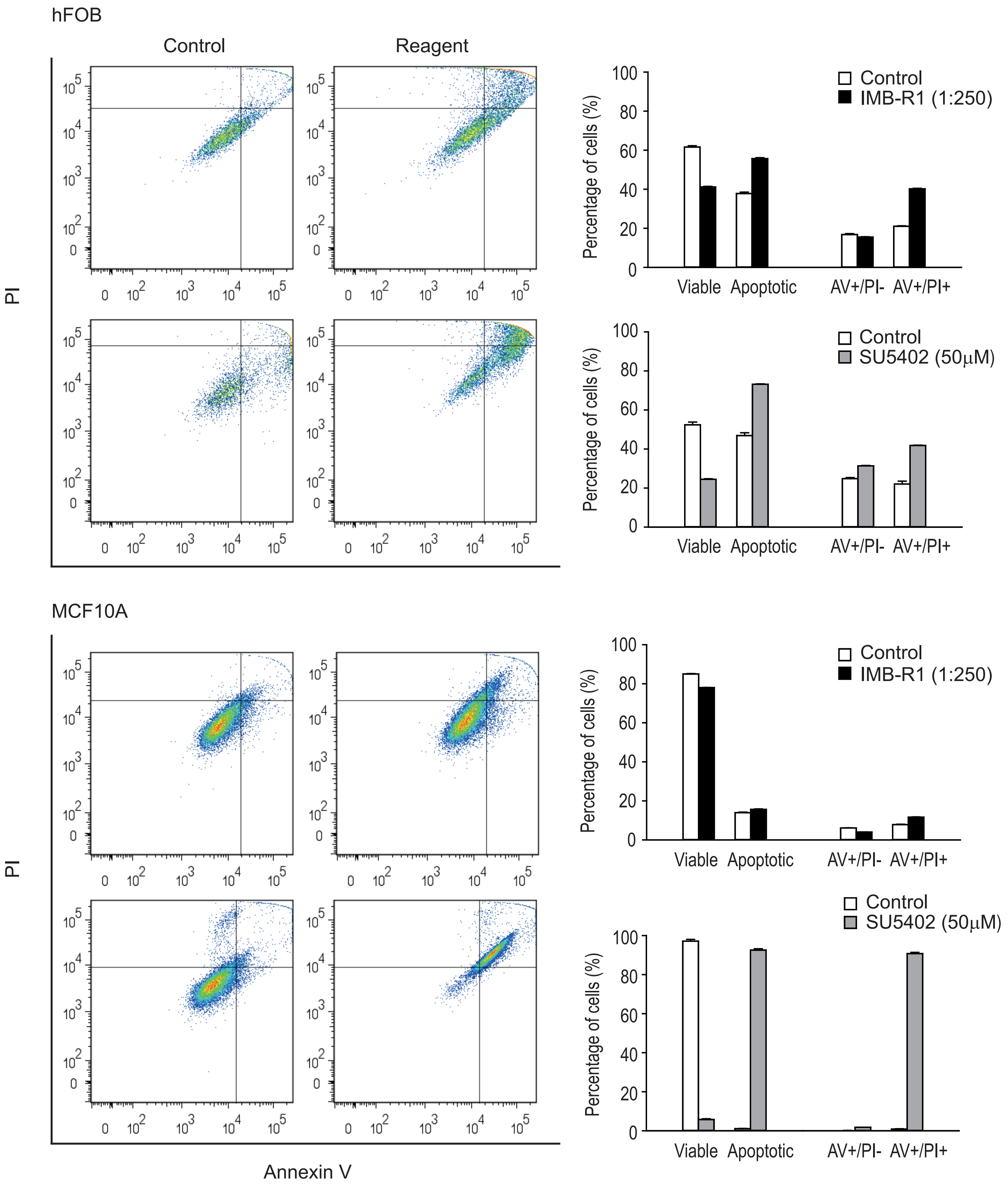

Supplementary Fig. 4

upregulated genes (gene symbol)

all 4 cells	hFOB&MG63*	MCF10A&MDAMB468*	2 normal cells*	2 cancer cells*
HMOX1	LOC100132564	AKR1B10	HMOX1	SPRY4
OKL38	DUSP6	OKL38	OKL38	DUSP6
GCLM	EIF5	CYP4F11	SPP1	EIF5
NQO1	PGF	LOC392437	SPANXA2	TMEM166
SRXN1	TSC22D1	PTGR1	LOC652683	TSC22D1
TXNRD1	TFRC	LOC344887	GCLM	FEN1
LOC642567	HMOX1	TXNRD1	LOC100133171	TFRC
PIR	PHTF1	ALDH3A1	SPANXB1	BRI3BP
PANX2	UCHL1	AKR1B15	SPANXB2	HMOX1
PHTF1	TXNIP	AKR1C2	NQO1	PHTF1
LOC731314	LOC731314	AKR1C4	SPANXA1	ORC6L
PGD	IL7R	GCLM	SPANXE	PDXK
ABHD3	UNKL	SRXN1	ANXA10	AKR1B10
C14orf149	PANX2	AKR1C3	SRXN1	PPP1R10
	GCLM	CES1	TXNRD1	UCHL1
	SERTAD1	NQO1	SLC7A11	ZC3HAV1
	SRXN1	LOC146909	LOC392437	LOC731314
	OBFC2A	SQSTM1	SQSTM1	LOC728006
	MAP1A	FTL	BAMBI	DDIT4L
	MARCKSL1	PIR	LOC642567	HNRPH3

downregulated genes (gene symbol)

all 4 cells	hFOB&MG63*	MCF10A&MDAMB468*	2 normal cells*	2 cancer cells*
SEPW1	GPX1	GPX1	SEPW1	PSG4
HDDC3	ATP6V1G2	SEPW1	KLF2	GPX1
GPX1	NIPSNAP1	SEPP1	HDDC3	DBP
TMEM205	HDDC3	CFD	EVL	SNORA8
SEPN1	RRAS	DHRS1	IQCK	ALDH6A1
DHRS1	ZNF25	SLC25A23	DBNDD2	KLHDC8B
NIPSNAP1	SEPW1	TMEM205	GPX1	HCFC1R1
SLC25A23	BBS2	ZNF467	MCM7	LOC729776
	HOPX	NIPSNAP1	ECHDC2	OSBPL7
	C11ORF74	LOC100132535	CTDSPL	DPM3
	AGBL5	HSD17B8	CBS	SSBP2
	GSTM2	BCAS4	ZFP90	EFEMP1
	SEPN1	SEPN1	TMEM205	NIPSNAP1
	SNX26	LOC339352	SRI	LOC339352
	CTSH	TNFSF12	SNRPN	HDDC3
	TMEM106B	DPM3	SEPN1	RRAS
	DBNDD2	HCFC1R1	NRBP2	CECR4
	CCDC24	LOC401397	DHRS1	ZNF25
	TADA1L	PC	LOC388588	SEPW1
	SLC25A23	MRPL41	DUT	CYP27A1

* Only top 20 genes are shown
